# Agreement Between Video-Based and In-Person Assessment in Patients with Knee Pain—A Prospective Repeated-Measures Pragmatic Study

**DOI:** 10.3390/jcm15093200

**Published:** 2026-04-22

**Authors:** Stefanos Karanasios, Athanasios Koutsouradis, Christina Mavrogiannopoulou, Vasiliki Sakellari, George Gioftsos

**Affiliations:** Laboratory of Advanced Physiotherapy (LadPhys), Physiotherapy Department, School of Health and Care Sciences, University of West Attica, 122 43 Egaleo, Greecevsakellari@uniwa.gr (V.S.); gioftsos@uniwa.gr (G.G.)

**Keywords:** tele-physiotherapy, musculoskeletal pain, knee, reliability, validity, assessment, diagnosis

## Abstract

**Background:** Digital health has accelerated telehealth uptake, yet evidence comparing video-based musculoskeletal assessment with traditional in-person examination is limited. This study evaluated the concurrent validity and interrater reliability of video-based physiotherapy assessment versus face-to-face assessment in patients with knee pain. **Methods:** Patients with knee pain underwent randomized consecutive in-person and video-based assessments by experienced musculoskeletal physiotherapists. Clinical diagnoses were categorized into seven groups (red flag, yellow flag, arthrogenic, tendinopathy, patellofemoral pain, muscle sprain, neurogenic). Primary outcomes were intermethod agreement and Cohen’s kappa; sensitivity, specificity, PPV, NPV, and interrater reliability for video assessments were also reported. **Results:** Forty-five participants (mean age 38 ± 6.5 years; 55.6% female) completed the study. In-person and video-based assessments produced identical diagnoses in 43/45 cases (Cohen’s κ = 0.92, *p* < 0.001). Telehealth accuracy was high across all diagnostic categories (90–100%). Interrater agreement between video-based assessors was 93.3% (κ = 0.89, *p* < 0.001). Agreement between assessments was moderately associated with KOOS (r = 0.312, *p* = 0.037). **Conclusions:** In this selected pragmatic sample, video-based physiotherapy assessment demonstrated high concurrent agreement and excellent interrater reliability with face-to-face assessment. Given the study’s sample size, repeated-measures design, and lack of an independent reference standard, these results indicate feasibility and intermethod agreement rather than diagnostic equivalence. Video assessment may be a feasible option for triage and management in selected settings, but further research in larger, more diverse populations and evaluation against independent reference standards is required.

## 1. Introduction

The World Health Organization (WHO) has actively pursued e-health initiatives and strategies for healthcare implementation during the last two decades [1]. Over time, e-health has evolved into the more comprehensive concept of digital health, which focuses on enhancing public health through the effective use of digital technologies [2]. The COVID-19 pandemic has further expedited this digital transformation, compelling healthcare systems worldwide to seek alternative solutions to traditional in-person care [3]. Recently, a significant number of patients have come to prioritize the accessibility and convenience offered by virtual care [4]. In this context, video-based consultations emerge as a viable and effective alternative to traditional face-to-face interactions, catering to the changing needs and preferences of patients [4,5]. Despite the strides in digital health implementation, virtual care applications remain in the early stages of development.

This context is particularly pertinent in the rehabilitation of musculoskeletal conditions such as knee pain, which is among the leading causes of pain and disability, significantly affecting a considerable portion of the adult population in many Western countries [6,7]. The lifetime prevalence of knee pain among adults is estimated to be around 50%, with a slightly higher incidence reported in women [6]. Clinical examinations typically encompass a comprehensive approach, including inspection, mobility assessment, strength evaluation, sensitivity testing, and palpation. Several clinical tests are utilized to assess and diagnose knee joint conditions, along with orthopedic tests targeting specific structures. Although these standardized clinical examinations have limitations, adapting these tests to a virtual context can still provide valuable insights [8].

Many patients with knee pain face significant challenges in accessing healthcare services, particularly in remote areas. In this context, video-based physiotherapy assessment and management may offer promising solutions by enhancing access to care [9,10]. Digital physiotherapy can provide several advantages over in-person rehabilitation, including overcoming geographical barriers, improving accessibility for homebound patients, reducing costs and travel time, and facilitating expert consultations through remote video conferencing [8,11]. Research evidence indicates that telerehabilitation can yield outcomes comparable to traditional in-person rehabilitation, making it an effective alternative for managing knee pain [12].

Despite several studies exploring various methods and approaches for video-based examinations, there remains insufficient research data regarding the agreement between diagnoses made via video-based assessments compared to traditional in-person clinical examinations [13,14,15]. A recent systematic review revealed that only two studies investigated the agreement between the two methods [8]. Although both reported relatively high agreement, their small sample sizes (18 and 14 patients) limit statistical precision and the ability to examine agreement across different diagnostic categories or severity levels [15,16]. Also, both investigations provide limited detail on specific examination procedures, test execution, examiner training, and the clinical information available to assessors. This lack of methodological transparency impedes replication and prevents determination of which components of the remote exam are reliable. Small, narrowly sampled studies are also vulnerable to selection bias and unlikely to capture the case mix (e.g., complex, irritable, or less common presentations) seen in routine practice. Critical steps to address these shortcomings include using a standardized examination protocol, reporting examiner training and blinding procedures, and assessing factors that influence diagnostic performance across clinically relevant subgroups.

Considering these gaps, our study aims to investigate the agreement between in-person and remote assessments, as well as the inter-tester reliability of video-based physiotherapy assessments, in patients with musculoskeletal knee conditions using standardized tests and clinical examinations.

## 2. Materials and Methods

### 2.1. Design

A repeated-measure, inter-rater agreement study was undertaken between June and September 2025. Participants with knee pain who had been referred for physiotherapy were invited to participate in the study. Written consent was obtained from all participants before their participation. Ethical approval to conduct this study was granted by the University of West Attica Research Ethics Committee (No 50429/02-06-2025).

### 2.2. Participants and Physiotherapists

A convenience sampling approach was undertaken, with participants recruited from a Physiotherapy Clinic (PhysioAthens) in Attica, Greece. To be eligible for inclusion, participants were required to be over 18 years of age and have their referrals classified from the triage as non-urgent for knee condition assessment.

Participants were excluded from the study if they reported any medical conditions that could compromise their safety during examination, such as significant cardiac or neurological diseases. Additionally, individuals with hearing or visual impairments that would hinder adequate participation in the telehealth assessment were not eligible. Furthermore, participants who were unable to mobilize independently—whether with or without a mobility aid—and those requiring the use of an interpreter were also excluded from the study.

The assessments were conducted by three physiotherapists (assessors), all of whom were postgraduate-qualified musculoskeletal physiotherapists with no prior experience in telehealth before this study. One assessor, CK, who had four years of experience, conducted all in-person assessments. Another assessor, AK, with 17 years of clinical experience, carried out the telehealth assessments. Subsequently, SK, who had 19 years of clinical experience, evaluated the recorded telehealth assessments. Immediately after the process, the third assessor evaluated the recorded telehealth assessments.

### 2.3. Sample Size

For calculating the required sample size, the G*Power software (version 3.1) was used. Given that the main objective of the study was to estimate the agreement between two diagnostic methods, with a categorical type of outcome variable, the statistical approach of Cohen’s Kappa index was chosen. Assuming an agreement of 0.60, a significance level (α) of 0.05, and a statistical power of 80%, it was determined that a sample of at least 45 participants was required [17].

### 2.4. Procedure

Participants who met the eligibility criteria and consented to join the study underwent two consecutive assessments, one in-person and one via telehealth, within the same visit. Both assessors were granted equal access to the participants’ medical records and radiological results. Following their initial assessment, if patients’ symptoms worsened, participants had the option to postpone the second assessment. The order of the assessments (in-person followed by telehealth or vice versa) was randomized for each participant. It was determined that both assessors would refrain from discussing the assessment results with the participant to prevent any possible confusion in the event of disagreements. To support the telehealth assessments, three pilot test subjects were recruited, providing the assessors with the opportunity to become acquainted with the videoconferencing platform and the required modifications for conducting physical examinations in this setting. The three assessors evaluated these subjects collaboratively, facilitating discussions regarding their observations.

#### 2.4.1. In-Person Assessment

During the in-person examination, standard procedures for physical assessment were followed, which included taking the patient’s medical history, palpation, evaluation of the range of motion, and specific diagnostic tests within a designated time frame of approximately 30 min. The assessment, encompassing both the patient interview and the physical examination, was pragmatic and adhered closely to established clinical practices [18].

#### 2.4.2. Video-Based Assessment

The video-based assessment was conducted using the Zoom platform. Participants were situated in a private room and were supplied with a tablet on a portable stand while the assessor (AK) was in a separate room within the same physiotherapy clinic, using a standard desktop computer connected to the building network. The room where participants were situated had a standard plinth, chair, towels, stepper, and small weights. Participants were interviewed by the assessor, who evaluated pain intensity, functional status and past medical history.

A structured video-based knee assessment protocol was implemented, including an initial screening and standardized patient positioning, followed by observational assessment of posture, alignment, swelling, gait, and functional symmetry. Functional testing comprised active range of motion (knee flexion/extension), single-leg balance, and squat performance (frontal and posterior views) to assess movement quality and load tolerance. Targeted clinical tests adapted for telehealth were then performed under guidance, including modified varus/valgus stress tests, squat assessment, modified Thessaly test, patellar tracking assessment, and Noble compression test, alongside patient-assisted palpation of key anatomical regions.

Where indicated, brief neurological screening (sensory and myotomal testing) was conducted. As conventional examination methods often required modification, participants were instructed to perform adapted tests independently (e.g., applying self-pressure for end-range assessment). Consistent with standard in-person clinical practice, the techniques selected were tailored to each patient’s presentation and their ability to safely perform the required tasks. A detailed description of the video-based assessment is provided in Supplementary Material File S1. The telehealth assessment protocol was informed by existing literature on video-based physical examination methods [15,19].

### 2.5. Outcomes

Patient demographic data were collected, including age, sex, occupation, duration of symptoms, pain intensity, weight, and height. Pain intensity was measured using the Numerical Rating Pain Scale (NRPS) from 0 (no pain) to 10 (worst pain). Functional status was evaluated using the Greek version Knee Injury and Osteoarthritis Outcome Score (KOOS), a comprehensive questionnaire that assesses symptoms, pain, and functional limitations [20].

#### Clinical Diagnosis

To examine the level of agreement between the video-based physiotherapy assessment and the in-person assessment in diagnosing knee patients, the following clinical diagnoses were established: D1 = Red flag (urgent medical care required), D2 = Yellow flag, D3 = Arthrogenic (osteoarthritis, rheumatoid arthritis, meniscal/ligament injuries, bursitis, plica syndrome, osteochondritis, or chondromalacia patellae), D4 = Tendinopathy (patellar, quadriceps, hamstring, iliotibial band or anserine tendinopathy), D5 = Patellofemoral pain syndrome, D6 = Muscle sprain, and D7 = Neurogenic (symptoms from nerve root or peripheral nerves caused by neural compromise or increased neural mechanosensitivity). Assessors were instructed to decide on the clinical diagnosis category based on the prominent factor affecting the patients’ presentation, while also considering the possibility of multiple factors influencing their clinical presentation. The assessors reviewed the same baseline clinical information and available investigations (medical records, prior imaging) before each assessment to reflect routine clinical practice. Examinations were conducted independently and assessors completed separate standardized assessment forms immediately after each encounter. No assessor had access to the other assessor’s ratings or completed forms until all data were locked.

### 2.6. Data Analysis

All data were analysed using the Statistical Package for the Social Sciences (SPSS), version 25 (IBM Corp., Armonk, NY, USA). Participant characteristics were described using percentages, frequencies, means with standard deviations (SD), or medians with interquartile ranges (IQR). To evaluate concurrent validity percentages, frequencies, sensitivity, specificity and Cohen’s kappa (κ) were calculated. The strength of agreement was assessed using a scale from 0 to 1, where scores from 0 to 0.20 reflect slight agreement, 0.21 to 0.40 indicate fair agreement, 0.41 to 0.60 represent moderate agreement, 0.61 to 0.80 signify substantial agreement, and scores from 0.81 to 1.00 indicate almost perfect agreement [21]. A multiclass confusion matrix was created, which was used to calculate the accuracy rates for each category (class-wise sensitivity/specificity) as well as the overall diagnostic accuracy. Interrater reliability was analyzed using the same statistical methods. Additionally, Spearman’s rho correlation coefficient was utilized, as it is suitable for non-normally distributed data, to examine whether there is a correlation between demographic and clinical variables (age, duration of symptoms, height, weight, pain intensity, KOOS) and the agreement between the in-person and telehealth assessors (Agreement-Total).

### 2.7. GenAI Usage Statement 

ChatGPT-4 (OpenAI, San Francisco, CA, USA) was used to improve the language, sentence structure, and grammatical clarity of this manuscript.

## 3. Results

**Figure 1:**
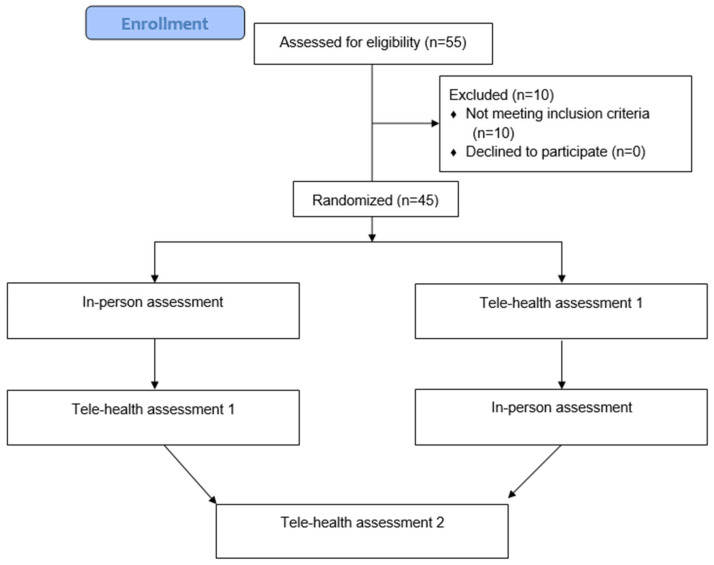
Flowchart of the recruitment and participation in the study.

Fifty-five individuals with knee pain volunteered to participate in the study. Three patients were excluded due to central nervous system impairments, 7 due to recent knee surgery. The remaining 45 participants met all inclusion criteria and provided written consent for their participation. All 45 individuals completed the study without any loss during the research period (Figure 1).

### 3.1. Participant Characteristics

Forty-five participants (25 women and 20 men) with a mean age (±SD) of 38 years (±6.5) were included in the study. None of them had previously experienced digital care. The median duration (IQR) of their symptoms was 10 weeks (4–64.5), while 29 of them (64.4%) worked as office workers. The mean pain intensity and disability were 4.24 (±2.11) and 76.3 (±12.38), respectively. The demographic characteristics of all included participants are presented in Table 1.

### 3.2. Agreement Between Video-Based and In-Person Assessment

The assessors reached the same diagnosis in 43 out of 45 patients (95.6%). The disagreements included one case where the in-person assessor considered it an arthrogenic disorder while the telehealth assessor identified a muscle sprain. In the other case, the in-person evaluator deemed it as a Patellofemoral Pain syndrome, where the remote evaluator considered it as an arthrogenic disorder. The agreement between in-person and video-based assessments was excellent (Cohen’s kappa = 0.92, *p* < 0.001). The cross-analysis between in-person and tele-health assessments is illustrated in Table 2.

The diagnostic accuracy of telehealth compared to in-person assessment was excellent for the diagnostic categories such as “red flag”, “tendinopathy”, and “neurogenic”, presenting absolute values (100%) for sensitivity, specificity, positive predictive value (PPV), and negative predictive value (NPV). Slight deviations were reported in the diagnosis of arthrogenic disorders (sensitivity 96%, specificity 95%) and patellofemoral pain (sensitivity 90%, specificity 100%). Muscle sprain demonstrated a sensitivity of 100% and specificity of 98%, with a slightly reduced PPV (69%) due to one false-positive case. Validity results are presented in Table 3. The mean (SD) duration of assessments was 28.9 (±6.1) and 37.8 (±7) minutes for in-person and video-based assessments, respectively.

### 3.3. Interrater Reliability

The agreement between video-based assessors was high (93.3%). Discrepancies occurred in three cases: two between tendinopathy and patellofemoral pain, and one between arthrogenic pain and patellofemoral pain syndrome. Inter-rater agreement measured by Cohen’s kappa was excellent (κ = 0.89, *p* < 0.001). The cross-tabulation of video-based assessments is shown in Table 4.

### 3.4. Secondary Outcomes

Correlation analysis showed that agreement between assessors was not significantly associated with participants’ age, sex, occupation type, symptom duration, height, weight, or pain intensity (*p* > 0.05) (Table 5). In contrast, there was a moderate positive correlation with the KOOS score (r = 0.312, *p* = 0.037), indicating that better knee function was associated with higher agreement in diagnostic assessment.

## 4. Discussion

The present study demonstrated substantial agreement between video-based and face-to-face physiotherapy assessments for patients with knee pain in a pragmatic clinical setting (physiotherapy clinic). Notably, the two assessment modalities produced identical diagnostic agreement in 95.6% of cases. These findings indicate that, notwithstanding the absence of hands-on examination, telehealth assessments can yield outcomes comparable to in-person evaluations. Similarly, the interrater reliability of video-based assessments suggested an excellent agreement between the assessors (93.3%). Our results substantiate the feasibility of implementing telehealth to improve access for patients with knee pain unable to attend in person.

The concurrent validity and interrater reliability of video-based assessment compared with in-person examination were high and consistent with previous reports investigating the agreement between the two methods in patients with knee pain [15,18,22]. Our findings were surprising because assessors had to identify multiple diagnostic categories rather than a single one (i.e., knee osteoarthritis) [22]. Patients were classified by the assessing physiotherapist into “possible” clinical diagnoses (red flag, yellow flag, arthrogenic, neurogenic, patellofemoral syndrome, tendinopathy, muscle sprain) based on standardized history-taking, symptom-reproduction tests, and test clusters. The categories were used as they align with management decisions and referral pathways and have established pragmatic validity in general musculoskeletal practice and in patients with knee pain [23,24,25]. Some diagnostic categories such as red/yellow flags and neurogenic nature included very few cases reflecting the typical case-mix seen in our physiotherapy clinic. Possibly, multicentre studies conducted in routine-care settings would help assess external validity and generalisability by capturing broader case-mix and less-standardised workflows. Physiotherapy decisions based on triage, conservative management and referral are generally driven by clinical presentation and risk stratification rather than definitive tissue pathology [26]. This emphasis on functional assessment may account for the higher agreement observed in our study (95.6%) compared to a prior one (67%) that used pathoanatomical diagnostic labels (e.g., medial collateral ligament strain with or without anterior cruciate ligament strain) [15]. Nevertheless, we acknowledge that broad categories reduce diagnostic specificity and may partially inflate intermethod agreement; therefore, results should be interpreted as agreement for pragmatic clinical classification rather than definitive diagnostic accuracy. Future studies should validate these categories against independent reference standards and evaluate agreement for more granular sign-based diagnoses.

The high diagnostic concordance observed in the present report is consistent with evidence that clinicians can accurately diagnose and triage common knee disorders using clinical assessment alone [27]. For instance, Décary et al. prospectively compared a physiotherapist’s musculoskeletal examination without imaging to expert physicians’ diagnoses with imaging in 179 patients and found excellent diagnostic agreement (k = 0.89, 95% CI 0.83–0.94) and good agreement for surgical triage (k = 0.73, 95% CI 0.60–0.86) [23]. Similar to our findings for video-based assessment, sensitivity and specificity for the physiotherapist’s examination for knee pathologies range from 82–100% and 96–100%, respectively, with favourable likelihood ratios, indicating strong diagnostic validity [27].

A careful consideration of our results should include the two instances (4.4%) in which diagnoses differed between modalities. In the first instance, a knee injury from a direct blow was classified as joint dysfunction by the in-person assessor but as a muscle sprain by the remote assessor; this discrepancy may reflect localized inflammation involving both joint and periarticular muscle tissues. This could reflect concurrent joint and periarticular myofascial involvement or localized sensitization, which may present differently to remote versus hands-on palpation [28]. Notably, the second remote assessor agreed with the in-person diagnosis. In the second instance, the in-person assessor diagnosed patellofemoral pain syndrome, whereas both video-based assessors recorded an arthrogenic dysfunction. Importantly, despite these diagnostic differences, the immediate short-term management, such as pain control, swelling reduction, and restoration of range of motion, would have been similar in both cases.

Video-based assessments demonstrated excellent inter-rater agreement (93.3%), with discrepancies in three cases: two between tendinopathy and patellofemoral pain, and one between arthrogenic pain and patellofemoral pain syndrome. Overlap in nociceptive sources and altered load distribution can blur boundaries between tendinopathy and patellofemoral pain, particularly when palpation is self-performed and sensory cues are limited [28]. Although telehealth technology may have some limitations, the close correspondence between inter-rater reliability and validity suggests that remaining discrepancies likely reflect patient performance variability, diagnostic overlap, and clinician reasoning and skill. Τhe diagnostic overlap between tendinopathy and patellofemoral pain can be amplified when subtle palpatory findings are less reliable, underscoring the need to integrate history, functional testing, and movement analysis alongside self-palpation. The study participants were asked to self-palpate anatomical structures for pain, swelling, and texture, with the online therapist prompting them to compare the sensations to those in the unaffected limb. This approach clearly extended the clinician’s sensory reach compared with inspection and active movement alone, but it introduced important limitations that likely contributed to some diagnostic uncertainty. Possibly, as previous reports suggested, targeted training and improved instructional design (shorter, anatomy-focused clips, live guided practice, or real-time feedback) could improve patient performance and reduce variability [15,29].

Framing knee pain as a multidimensional condition where structural pathology interacts with myofascial, peripheral and central sensitization, motor control alterations, and psychosocial factors helps interpret our results in pragmatic clinical classification rather than definitive tissue-based diagnoses [30]. Many knee pain presentations reflect overlapping nociceptive and non-nociceptive mechanisms (e.g., local tendon or myofascial nociception, altered loading patterns, and pain sensitization), which can produce similar clinical signs but require different mechanistic emphasis in management [28,30,31]. This perspective may explain the few discrepancies observed (e.g., arthrogenic versus myofascial/muscle sprain or tendinopathy versus patellofemoral pain), since subtle palpatory cues and mechanistic distinctions are harder to capture remotely. Growing evidence indicates that integrating brief mechanistic screening items such as pain sensitization questions, movement-provocation clusters and, where available, real-time feedback with objective adjuncts (i.e., wearable motion and force sensors, standardized photos and videos) could enhance remote assessment fidelity and guide targeted conservative management in patients with knee and low-back pain [32,33,34]. Such technological advancements may also be valuable in cases with diagnostic overlap, such as tendinopathy and patellofemoral pain, by improving the objectivity and reliability of video-based assessments.

An important consideration of the present study is that the face-to-face assessments were conducted by a relatively less experienced physiotherapist, whereas the video-based assessments were performed by more experienced clinicians. This discrepancy may have influenced the observed level of agreement, given that prior evidence suggests clinical experience plays a key role in telehealth assessment, particularly through enhanced pattern recognition and clinical reasoning [35,36]. Experienced physiotherapists may be better able to compensate for the absence of physical contact by relying on detailed history-taking, movement analysis, and targeted questioning [35]. Consequently, it remains unclear whether similar levels of agreement would be achieved if video-based assessments were conducted by less experienced clinicians.

Our sample was relatively small consisted of forty-five middle-aged to young population with acute/sub-acute symptoms and low disability scores which may limit the generalizability of the findings to older patients or those with more severe or complex knee conditions. As such, caution is warranted when extrapolating these results to populations with advanced pathology, higher pain levels, or significant functional impairment. Notably, the moderate positive correlation between disability scores and validity indicates that participants with better self-reported knee function produced more consistent diagnostic assessments. Possibly, it may be easier to assess remotely patients with higher function, because they can follow instructions more accurately, tolerate testing better, and produce clearer movement patterns [37,38]. At the same time, those with greater dysfunction or longer symptom duration may present more complex or inconsistent signs that reduce agreement between the two modalities. It would be valuable to stratify patients for remote assessment, favoring those with milder dysfunction or shorter symptom duration and also, building in longer appointment slots for complex cases. A structured pre-assessment familiarization or brief training protocol for patients with knee pain (e.g., standardized instructions or demonstration videos) with or without caregiver assistance may further improve the consistency of test performance and reduce variability in video-based assessments, particularly for movements requiring precise execution or patient-assisted palpation.

In line with previous reports, online examinations in our study lasted longer than face-to-face assessments because clinicians needed extra time to explain positioning, coach self-application of modified tests, and qualify participants’ findings; this has service-delivery implications for scheduling, clinician workload, and patient fatigue [39,40]. Longer appointments for more complex cases, combined with optional follow-up video sessions, may reduce patient stress and improve assessment accuracy. Nevertheless, this study was not pre-specified as a pilot or feasibility trial; therefore, formal feasibility metrics (pre-defined progression criteria, sample-size justification for feasibility, and structured acceptability measures) were not collected, and findings should be interpreted as preliminary and hypothesis-generating to inform future pilot/feasibility work.

Other study limitations that should be considered include the repeated-measures design that may have allowed participants to learn from the initial examination, and subsequently, it may have affected the findings on the subsequent assessment, increasing the agreement between the assessors [15,41]. However, symptom provocation between sessions could have also changed the clinical presentation in irritable or latent cases, a phenomenon that may have affected agreement between the two methods. Possibly, longer washout intervals between assessments may have decreased this source of bias. It should also be acknowledged that the use of the same visit, shared clinical information, and prior discussion of pilot cases may have introduced a degree of standardisation that does not fully reflect routine clinical conditions, thereby potentially limiting external validity.

Future research should evaluate the validity of the telehealth assessment, including wider age ranges and chronic conditions. Also, the evaluation of strategies aiming to improve video-based assessment accuracy, such as pre-visit training, caregiver-assistance, standardized instructional media, pressure- or motion-sensing adjuncts, is required. Possibly, larger samples will allow further subgroup analyses by age, socioeconomic status, and geographical representation to clarify which patients with knee pain are most appropriate for telehealth musculoskeletal assessment.

## 5. Conclusions

This prospective repeated-measures study demonstrates high agreement and interrater reliability between video-based and in-person physiotherapy assessments in a selected population of patients with knee pain. These results support the potential utility of telehealth for triage and management decisions in selected pragmatic clinic settings. However, agreement alone does not establish equivalence in diagnostic accuracy, treatment decisions, or clinical outcomes. However, given the limited sample size, the potential bias introduced by the repeated-measures design, and the absence of an independent reference standard, these findings should be interpreted as evidence of feasibility and intermethod agreement rather than diagnostic equivalence. Further research in larger, more diverse populations including studies using independent reference standards and measuring downstream clinical outcomes is needed to determine the broader clinical applicability of video-based assessment.

## Figures and Tables

**Table 1:** Participants’ demographic and characteristics at baseline (N = 45).

Women	25 (55.6%)
Men	20 (44.4%)
Age, mean (SD), years	38 (6.5)
Height, cm mean (SD)	171 (8.6)
Weight, kg mean (SD)	73.9 (14.3)
BMI, kg/cm^2^ mean (SD)	25.3 (3.1)
Symptom duration, weeks median (IQR),	10 (4–64.5)
Office worker	29 (64.4)
Other	16 (35.6)
KOOS, mean (SD)	76.3 (12.3)
Worst Pain Past Week, mean (SD)	4.24 (2.1)

Values are presented as numbers (percentage), unless otherwise indicated. Abbreviations: SD, standard deviation; IQR, interquartile range; KOOS, Knee Injury and Osteoarthritis Outcome Score; BMI, Body Mass Index.

**Table 2:** Cross-analysis agreement between in-person and video-based assessments.

		Agreement (95.6%)							Disagreement (4.4%)
		Telehealth Assessment							
		D1	D2	D3	D4	D5	D6	D7	
In-person Assessment	D1	1	0	0	0	0	0	0	0
	D2	0	0	0	0	0	0	0	0
	D3	0	0	25	0	0	1	0	1
	D4	0	0	0	5	0	0	0	0
	D5	0	0	1	0	9	0	0	1
	D6	0	0	0	0	0	2	0	0
	D7	0	0	0	0	0	0	1	0

Abbreviations: D1 = Red flag (urgent medical care required), D2 = Yellow flag, D3 = Arthrogenic (osteoarthritis, rheumatoid arthritis, meniscal/ligament injuries, bursitis, plica syndrome, osteochondritis, or chondromalacia patellae), D4 = Tendinopathy (patellar, quadriceps, hamstring, iliotibial band or anserine tendinopathy), D5 = Patellofemoral pain syndrome, D6 = Muscle sprain, and D7 = Neurogenic.

**Table 3:** Agreement of video-based compared to in-person assessment.

Clinical Diagnosis	TP	FN	FP	TN	Sensitivity	Specificity	PPV	NPV
Red flag	1	0	0	44	1.00	1.00	1.00	1.00
Arthrogenic	25	1	1	18	0.96	0.95	0.96	0.95
Tendinopathy	5	0	0	40	1.00	1.00	1.00	1.00
Patellofemoral Pain	9	1	0	35	0.90	1.00	1.00	0.97
Muscle sprain	2	0	1	42	1.00	0.98	0.69	1.00
Neurogenic	1	0	0	44	1.00	1.00	1.00	1.00

Abbreviations: TP, True Positive; FN, False Negative; FP, False Positive; TN, True Negative; PPV, Positive Predictive Value; NPV, Negative Predictive Value.

**Table 4:** Cross-analysis agreement between the two video-based assessments.

		Agreement (93.3%)							Disagreement (6.7%)
		Telehealth Assessment							
		D1	D2	D3	D4	D5	D6	D7	
In-person Assessment	D1	1	0	0	0	0	0	0	0
	D2	0	0	0	0	0	0	0	0
	D3	0	0	26	0	0	1	0	1
	D4	0	0	0	4	1	0	0	1
	D5	0	0	0	1	8	0	0	1
	D6	0	0	0	0	0	2	0	0
	D7	0	0	0	0	0	0	1	0

Abbreviations: D1 = Red flag (urgent medical care required), D2 = Yellow flag, D3 = Arthrogenic (osteoarthritis, rheumatoid arthritis, meniscal/ligament injuries, bursitis, plica syndrome, osteochondritis, or chondromalacia patellae), D4 = Tendinopathy (patellar, quadriceps, hamstring, iliotibial band or anserine tendinopathy), D5 = Patellofemoral pain syndrome, D6 = Muscle sprain, and D7 = Neurogenic.

**Table 5:** Correlation analysis between demographic and baseline participant characteristics and agreement between in-person and video-based.

Variable	Correlation	*p*-Value
Sex	χ^2^ = 1.67	0.196
Age	r = 0.208	0.171
Height	r = −0.075	0.625
Weight	r = −0.067	0.664
Symptom duration	r = 0.28	0.063
Occupation type	χ^2^ = 1.155	0.283
Worst Pain Past Week	r = 0.147	0.336
KOOS	r = 0.312	0.037

Notes: Values are presented as numbers (percentage), unless otherwise indicated. Abbreviations: KOOS, Knee Injury and Osteoarthritis Outcome Score.

## Data Availability

The raw data supporting the conclusions of this article will be made available by the authors upon request.

## Supplementary Materials

The following supporting information can be downloaded at: https://www.mdpi.com/article/10.3390/jcm15093200/s1.

## Abbreviations

The following abbreviations are used in this manuscript:

WHO	World Health Organization
NRPS	Numerical Rating Pain Scale
KOOS	Knee Injury and Osteoarthritis Outcome Score
SPSS	Statistical Package for the Social Sciences
IQR	Interquartile Range
SD	Standard Deviation
BMI	Body Mass Index
PPV	Positive Predictive Value
NPV	Negative Predictive Value
