# Supplementary material for: Agreement Between Video-Based and In-Person Assessment in Patients with Knee Pain—A Prospective Repeated-Measures Pragmatic Study
Source: J Clin Med. 2026 Apr 22;15(9):3200. doi: 10.3390/jcm15093200 (PMC13164119; doi:10.3390/jcm15093200)
Supplement: Supplementary file 1 [file jcm-15-03200-s001.zip › jcm-4250571-supplementary.pdf]

**Overview:**

- Are you in pain right now? YES □ NO □ If yes, on a 0–10 scale how much pain have you had in the past week? ____/10
- Are you okay to proceed? YES □ NO □

**Positioning / Setup**

- Please sit along the white line on the bed with your body facing the camera.

**Observation (standing/sitting)**

1) Does the patient stand symmetrically? (Check knee alignment for varus/valgus) YES □ NO □
   If no, explain: ________________________________________________
2) Is the RIGHT □ and/or LEFT □ knee swollen?
3) Is there an antalgic gait? YES □ NO □
4) Is there an antalgic posture when sitting? YES □ NO □
5) Is there obvious deformity? YES □ NO □
6) Are limb positions symmetrical? YES □ NO □
7) Is the pelvis neutral? YES □ NO □ If no, why: _____________________
8) Are soft tissues symmetrically distributed? YES □ NO □
9) Is there obvious muscle weakness? YES □ NO □
10) Any scars suggesting recent injury or surgery? YES □ NO □
11) During joint movement is there crepitus, catching, or unusual sound? YES □ NO □ If yes, describe:
   ________________________________________________

**Clinical assessment (examine the healthy knee first, then the painful knee)**

- Does one knee feel warmer? (patient checks with back of hand)

YES □ NO □ If yes: RIGHT □ LEFT □ BOTH □

***Gait assessment***

- Ask patient to walk along the room and evaluate:
  - Asymmetry in weight-bearing time on the affected leg? YES □ NO □
  - Normal loading range during stance? YES □ NO □
  - Normal swing of the affected limb? YES □ NO □
  - Hip abductor weakness on the affected side (Trendelenburg gait)? YES □ NO □
  - On the healthy side? YES □ NO □
  - Does the patient lean to one side overall? YES □ NO □ If yes, note additional observations: ________________________

***Neural tissue sensitivity and sensory testing***

- Straight Leg Raise (SLR) nerve sensitivity: Positive? YES □ NO □
- Sensory testing (only if neurological symptoms present). Use a cotton swab to test corresponding dermatomes:
    - L3: area above medial patella. Normal R/L □ Abnormal R/L □
    - L4: area lateral to the big toe (first MTP). Normal R/L □ Abnormal R/L □
    - L5: area between 1st and 3rd toes. Normal R/L □ Abnormal R/L □
    - S1: lateral to 5th toe. Normal R/L □ Abnormal R/L □
    - S2: plantar surface excluding heel. Normal R/L □ Abnormal R/L □

Myotome testing (only if neurological symptoms present)

- Evaluate visible or palpable muscle contraction via camera.
    - L3 (iliopsoas / T12-L3): from sitting ask patient to lift knee up. Right: none □ mild □ strong □ Left: none □ mild □ strong □
    - L4 (adductors / L2-L4): from side ask to bring lower leg upward (or from sitting abduct then adduct). Right: none □ mild □ strong □ Left: none □ mild □ strong □
    - L5 (extensor hallucis longus): ask patient to lift toes. Right: none □ mild □ strong □ Left: none □ mild □ strong □
    - S1 (gastrocnemius / S1): supine, ask for plantarflexion (push as if on gas). Right: none □ mild □ strong □ Left: none □ mild □ strong □
    - S2 (intrinsic foot muscles / toe flexors): supine, ask to flex toes. Right: none □ mild □ strong □ Left: none □ mild □ strong □

**Clinical tests (video-guided instructions and expected observations)**

Palpation along joint line (patient or helper palpates along joint line).

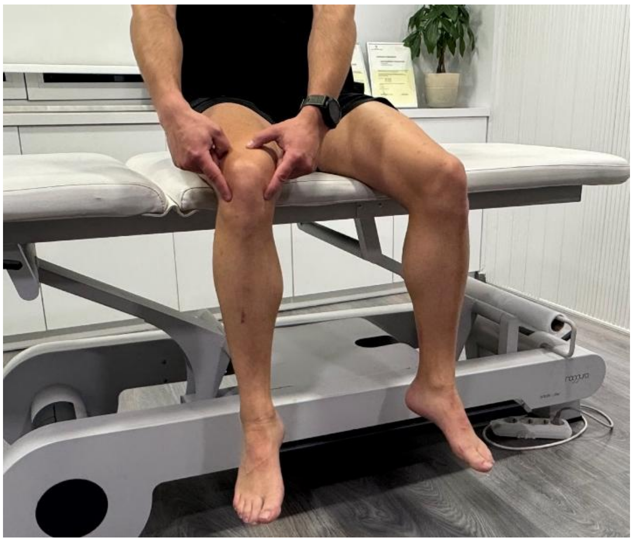

Pain/reproduction of symptoms suggests possible osteoarthritis, patellar tendinopathy, or proximal tibiofibular injury.

- Pain? YES □ NO □

  Patellar tracking:

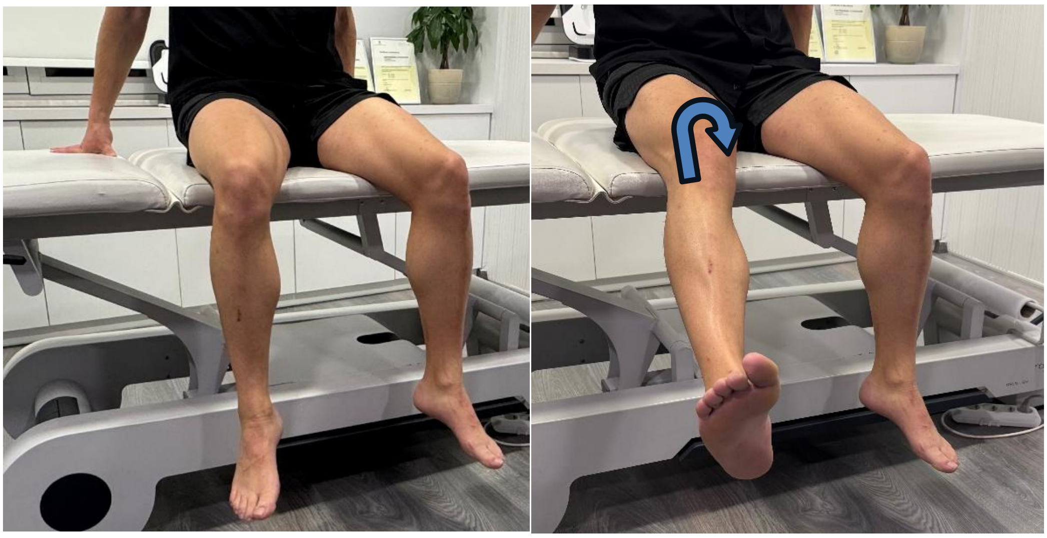

During knee extension the patella should follow a smooth arc. A J-sign indicates patellar instability.

- J-sign present? YES □ NO □

Active knee extension/flexion sitting:

A. B.

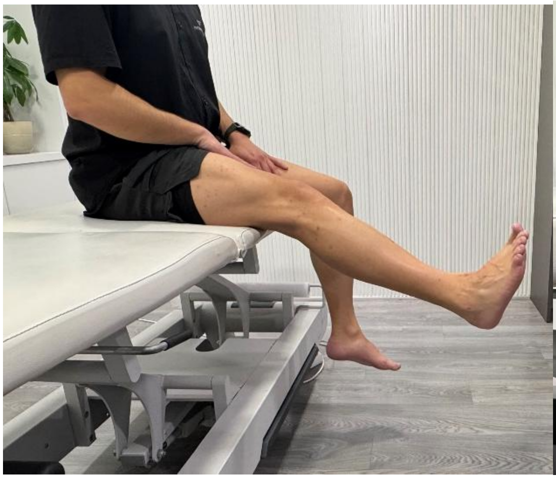

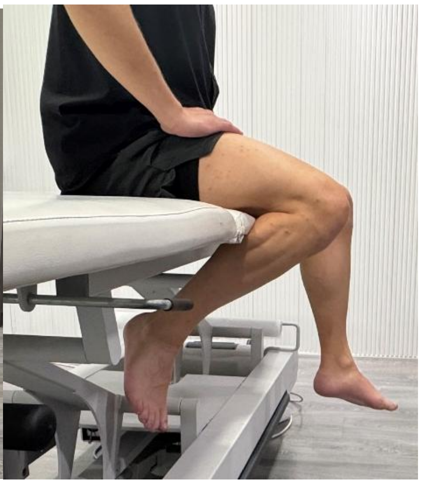

- Show patient sitting; have them demonstrate: A: full knee extension B: knee flexion
- Range of motion (extension and flexion), comment: ____________________
- Can they put full weight on the painful knee? YES □ NO □
- Single-leg balance? YES □ NO □ If yes, how many seconds? ____

Squat with full weight bearing (frontal and posterior views)

A: 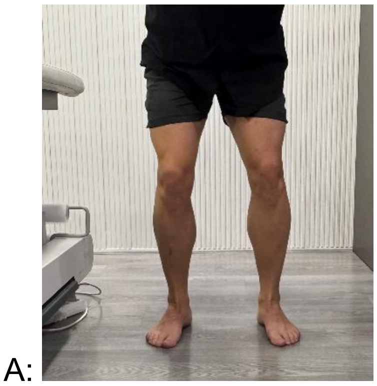 B: 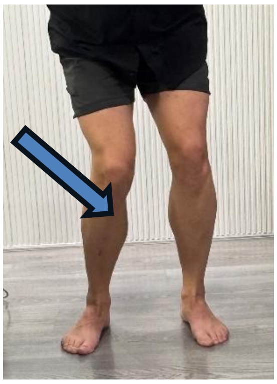

A: smooth controlled descent (front view) B: medial collapse of the knee during descent (arrow) with pain suggests involvement of the medial collateral structures and weakness of the involved limb.

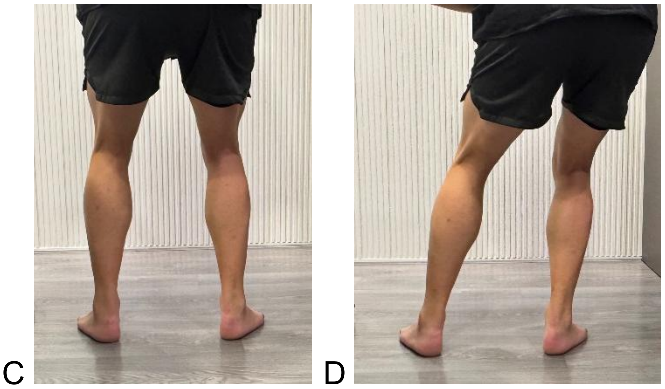

C D

C: smooth controlled descent (rear view) D: hip shift during descent (arrow) suggests weakness or restricted motion on the side of shift.

Medial collateral involvement/limb weakness? YES □ NO □

Weakness or ROM restriction on side of hip shift? YES □ NO □

<u>Varus/valgus (medial and lateral collateral) stress (modified)</u>

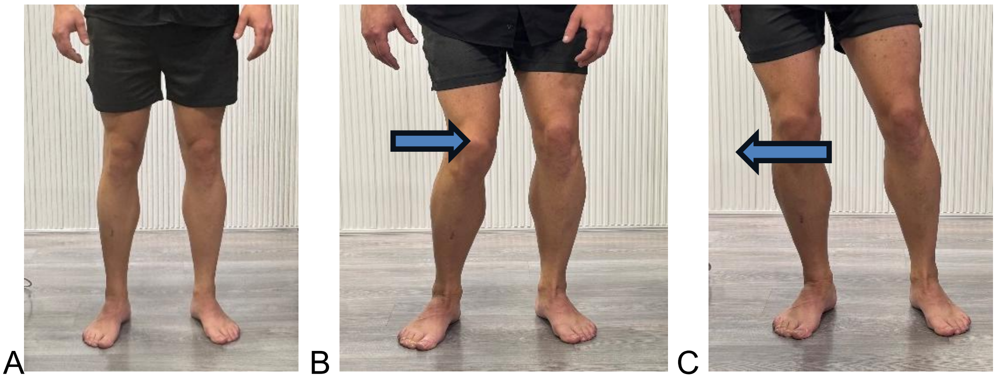

A B C

A: neutral standing

B: modified valgus maneuver — instruct patient to bend knee inward

C: modified varus maneuver — instruct patient to bend knee outward

Pain with either maneuver suggests ligamentous involvement on the stressed side. Lateral collateral ligament injury? YES □ NO □

Medial collateral ligament injury? YES □ NO □

Modified Thessaly (single-leg twist) — caution in elderly

Pain with twisting on a single limb while rotating? YES □ NO □

If positive, note possible meniscal injury: ____________________

Modified Noble compression test

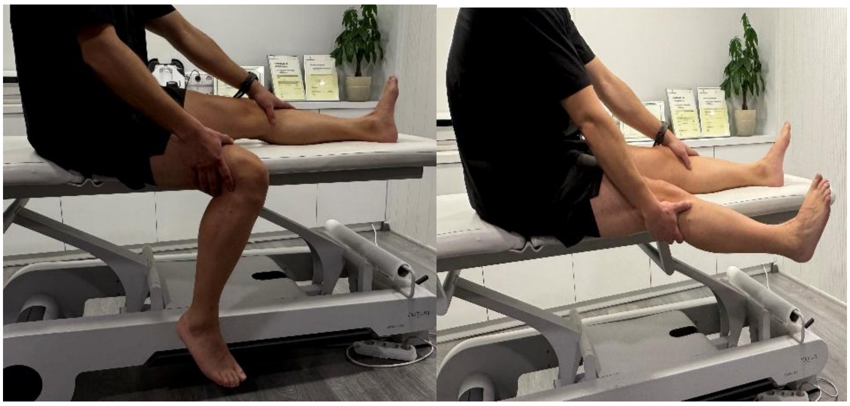

Instruct patient to press on distal iliotibial band region then extend the knee. Pain suggests IT band syndrome or fibular head dysfunction.

IT band syndrome / fibular head dysfunction? YES □ NO □

Patellofemoral cartilage (crepitus) test

Patient places thumbs over patella, contracts quadriceps. Pain indicates positive test.

Pain? YES □ NO □

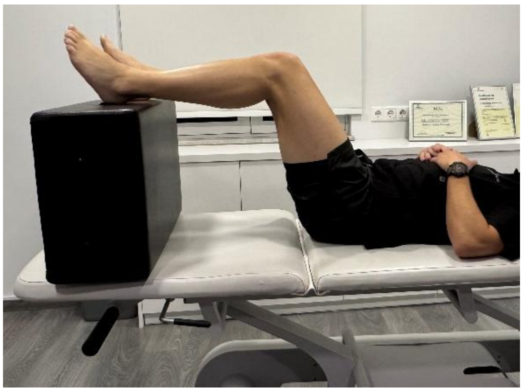

Posterior sag sign

- Supine with knees ~90° supported under heels. A notable difference in tibial height indicates posterior cruciate ligament involvement.
- PCL involvement? YES □ NO □

Modified Thomas test

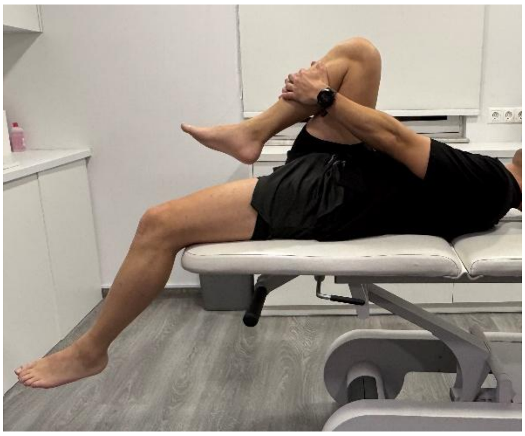

Instruct patient to pull the unaffected knee to chest and lie back.

- If the tested knee is extended → possible rectus femoris involvement.
- If the hip remains flexed → possible iliopsoas involvement.
- Rotation → possible IT band restrictions.
- Hip abduction → possible tensor fasciae latae/IT band source of pain.
- Any of the above present? ___________________________________

Passive palpation at pes anserinus insertion — pain? YES □ NO □

Passive palpation in popliteal fossa — Baker's cyst present? YES □ NO □

Active isometric quadriceps at 90° knee flexion (opposed by other leg): pain indicates quad injury/weakness. Pain? YES □ NO □

Active isometric hamstrings at 0° (patient presses foot into bed base): pain indicates hamstring injury/weakness. Pain? YES □ NO □

Notes for video administration

- Instruct the patient to position camera to show full limb movements and frontal/posterior views as required.
- Ask the patient to perform movements slowly and repeat when necessary.
- If any neurological deficit or severe pain is suspected, advise in-person assessment.
